# Relationship between microbiota awareness, nutrition literacy, and health literacy among adolescents

**DOI:** 10.55730/1300-0144.5871

**Published:** 2024-08-05

**Authors:** İzzet ÜLKER, Muhammet Ali AYDIN, Metin YILDIZ, Gönül GÖKÇAY, Ayşe ELKOCA, Mehmet Salih YILDIRIM, Abraham TANIMOWO, Dursun Alper YILMAZ

**Affiliations:** 1 Department of Nutrition and Dietetics, Division of Nutrition and Dietetics, Faculty of Health Sciences, Erzurum Technical University, Erzurum, Turkiye; 2 Department of Nursing, Division of Public Health Nursing, Faculty of Health Sciences, Erzurum Technical University, Erzurum, Turkiye; 3 Department of Midwifery, Division of Midwifery, Faculty of Health Sciences, Sakarya University, Sakarya, Turkiye; 4 Department of Nursing, Division of Public Health Nursing, Faculty of Health Sciences, Kafkas University, Kars, Turkiye; 5 Department of Midwifery, Division of Midwifery, Faculty of Health Sciences, Gaziantep Islam Science and Technical University, Gaziantep, Turkiye; 6 Department of Nursing, Division of Public Health Nursing, Faculty of Health Sciences, Ağrı İbrahim Çeçen University, Ağrı, Turkiye; 7 Department of Child Health, Division of Child Health, College of Medicine, University of Ibadan, Oyo, Nigeria

**Keywords:** Adolescent, health literacy, microbiota awareness, nutrition literacy

## Abstract

**Background/aim:**

Microbiota awareness, nutritional literacy, and health literacy levels in adolescents have a significant impact on their health and well-being. This research was conducted to examine the relationship between microbiota awareness, nutrition literacy, and health literacy in adolescents.

**Material and methods:**

This research was structured with a descriptive-correlational design. The study population comprised adolescents aged 10–19 years, living in Türkiye (n = 739), between June 2022 and February 2024. Data were analyzed using SPSS 22.0, G*Power 3.1, and R programming language 4.1.3.

**Results:**

The total effect of the health literacy variable on nutritional literacy was 0.2311, and this was statistically significant at a 95% confidence interval (CI) (p < 0.05). In terms of the health literacy variable, the direct effect of the nutrition literacy variable on the microbiota awareness variable was 0.2888, and this was statistically significant at the 95% CI (p < 0.05). In terms of the nutritional literacy variable, the direct effect of the health literacy variable on the microbiota awareness variable was 0.1707, and this was statistically significant at the 95% CI (p < 0.05). Nutrition literacy had a partial mediating role in the effect of health literacy on microbiota awareness (lower limit CI: 0.045; upper limit CI: 0.0894). The most accurate prediction of machine learning approaches to predict microbiota awareness was made with random forest with shapley additive explanations values, and the most important variable that should be in the model to predict the microbiota awareness variable was the nutrition literacy variable.

**Conclusion:**

Microbiota awareness increased as health literacy and nutrition literacy increased. In the machine learning approach prediction, the most important variables affecting microbiota awareness were health literacy and nutritional literacy. Longitudinal studies on microbiota awareness are recommended.

## 1. Introduction

The human body hosts a complex assembly of bacteria, viruses, fungi, and other unicellular organisms, collectively known as the microbiota. This community begins to form immediately after birth, quickly evolving into a dynamic ecosystem. In the initial two to three years of life, bifidobacteria emerge as a predominant force, playing a key role in stabilizing this microbiome diversity [1,2]. Over time, the microbial makeup of human adults becomes more diverse and rich, encompassing several hundred species-level phylotypes, primarily dominated by phyla Bacteroidetes and Firmicutes [2,3]. It has been theorized that the formation of bacterial ecosystems during early childhood affects the microbial makeup and vulnerability to diseases for a lifetime [2]. Dietary habits have been shown to modify the microbiota composition, with malnutrition leading to a reduction in the number of Bacteroidetes, known for their ability to break down carbohydrates present in high-calorie Western foods. Additionally, alterations in the microbiome have been associated with diet-induced conditions like allergies and obesity [4].

Health literacy is a critical component of health promotion that has received increased attention since the 1990s. The World Health Organization (WHO) describes health literacy as achieving a level of knowledge, personal abilities, and confidence essential for initiating actions aimed at improving individual and community health by transforming personal lifestyles and living conditions. It represents a strategy for activating health information to empower individuals [5]. Inadequate health literacy has been linked to poor management of chronic conditions such as cardiovascular disease [6,7], asthma [8,9] and diabetes [10,11], as well as increased morbidity and mortality. Health literacy is critical for promoting healthy nutrition practices. Appropriate nutrition contributes to the advancement of general welfare, hence improving one’s quality of life [12]. Additionally, a proper diet is related to improved disease management [13]. Individuals with low health literacy face difficulties comprehending and interpreting dietary information [14]. Nutrition literacy enables us to overcome this barrier.

Nutrition literacy is the ability to access, interpret, understand, and use nutritional information and knowledge effectively to make informed and healthy dietary choices, which is linked to maintaining overall healthy diets and the quality of one’s diet [15,16]. Additionally, nutrition literacy is a subset of health literacy; in adolescents, low health literacy is connected with obesity [17] and lower levels of health-promoting activities [18–20]. Inadequate nutrition knowledge and abilities contribute to an individual’s susceptibility to excessive weight gain and the accompanying health problems [20]. Adolescents’ health literacy demonstrates their capacity to meet expanding health needs, as it is associated with numerous aspects of health promotion, including food.

Adolescence provides a window of opportunity for a successful transition to adulthood. The microbial composition, nutrition literacy, and health literacy levels at this age have a significant impact on the adolescent’s health and welfare, as well as intergenerational health outcomes [21]. Promoting healthy eating behaviors in adolescents is critical for proper growth and development, disease prevention, overweight and obesity prevention, and the establishment of sustainable healthy eating patterns in adulthood [22]. Although few studies have been conducted in Türkiye on microbiota awareness, nutrition literacy, and health literacy in adolescents, the current study aimed to ascertain the association between microbiota awareness, nutrition literacy, and health literacy in adolescents.

This study is important because it deals with microbiota awareness, which is important in the developmental period of adolescents, and since there have been no studies addressing the relationship between health literacy and nutrition literacy, it will shed light on both the literature and intervention studies to be conducted with adolescents.

### Research hypotheses (H)

2. H_1_: An adolescent’s health literacy level affects their nutritional literacy level.
3. H_2_: An adolescent’s health literacy level affects their microbiota awareness level.
4. H_3_: An adolescent’s nutrition literacy level affects their microbiota awareness level.
5. H_4_: Nutritional literacy has a mediating role in the effect of an adolescent’s health literacy level on their microbiota awareness level.

## 2. Materials and methods

### 2.1. Participants

The research was conducted using a descriptive and correlational design. The study population consisted of 12,793,612 adolescents aged 10–19 years who were registered in the Turkish Statistical Institute Address Based Population Registration System’s, province, age group, and sex data system^1^. The sample size was calculated as 384 using a known sampling method. This study included 739 individuals between 10 and 19 years of age. Based on the results obtained from these participants, post hoc power analysis was conducted, and the power of the study was calculated as 100% at a 95% confidence interval (CI) for a medium effect size [23]. The Strengthening the Reporting of Observational Studies in Epidemiology guidelines were used to report this research article [24].

Place and time of the research: In Türkiye between June 2022 and February 2024.

Inclusion criteria: Being 10–19 years of age and voluntarily agreeing to participate in the study.

Exclusion criteria: Not agreeing to participate in the study or leaving the study prior to its completion.

### 2.2. Data collection tools

#### Introductory Information Form

This form was created by the researchers and consists of questions related to the individuals’ demographic information.

#### Microbiota Awareness Scale

The scale was developed by Külcü and Önel in 2020 [25]. It is a five-point Likert type scale and consists of 20 items, including 18 positive and two negative questions. The Microbiota Awareness Scale has four subfactors:

General Information subdimension (6 items: 1, 2, 4, 5, 6, 13)
Product Information subdimension (4 items: 17, 18, 19, 20)
Chronic Disease subdimension (5 items: 8, 10, 12, 14, 16)
Probiotic and Prebiotic subdimension (5 items: 3, 7, 9, 11, 15).

The scale’s internal consistency was calculated using Cronbach’s alpha and was determined as 0.852. An increase in the score indicates an increased awareness of the microbiota. In the current study, the Cronbach alpha value was 0.86.

#### Adolescent Nutrition Literacy Scale

It was developed by Bari [26] in 2012 to determine the nutritional literacy of adolescents. The validity and reliability study in Turkish was conducted by Türkmen et al. [27] in 2017. The scale consists of 22 items and three subdimensions. An increase in the scores indicates an increase in the level of nutritional literacy. In the validity and reliability study of the scale, the Cronbach alpha value was reported as 0.80 [27]. The Cronbach’s alpha value for the current study was 0.89.

#### Health Literacy Scale

The scale, which determines an individual’s health literacy level, was developed by Sørensen et al. [28]. Aras and Temel [29] validated and established its reliability in Turkish in 2017. The scale consists of 25 items and four subdimensions. The minimum score for the whole scale is 25 and the maximum is 125. Low scores indicate inadequate, problematic, and weak health literacy status, while high scores indicate adequate and very good. As the score obtained from the scale increases, the health literacy level of the individual also increases. The Cronbach’s alpha coefficient of the original scale was 0.95, and the Cronbach’s alpha coefficient determined for the scale subdimensions ranged between 0.90 and 0.94. The Cronbach’s alpha value for this study was calculated as 0.93.

### 2.3. Data collection

The data collection process was conducted online, whereby participants were required to provide their consent to participate in the study after being presented with the study’s objectives through an online form.

### 2.4. Data evaluation

The collected data were analyzed using the SPSS statistical package program. The normality of the data was assessed using appropriate tests, and the normality of the data distribution was determined based on the kurtosis and skewness values falling within the acceptable range of −1.5 to 1.5 [30]. Furthermore, data analysis was performed using process analysis, and p < 0.05 was considered statistically significant. For the estimation of the model, R programming language 4.1.3 was used. Machine learning offers many advantages over regression analysis and stands out as a more powerful approach to solving a variety of problems. The strengths of machine learning over regression are given below:

1. Coping with Complexity: While machine learning algorithms can handle multidimensional and complex data sets, traditional regression analysis can struggle to deal with such data. Machine learning can make effective predictions even with high dimensionality and nonlinear relationships.
2. Flexibility: Machine learning can model nonlinear relationships and automatically discover hidden patterns and relationships in the data. This provides the ability to solve a wider set of problems compared to the linear relationships assumed by regression models.
3. Automatic feature selection: Some machine learning methods can automatically select important features and ignore less important ones. This improves the generalization of the model while reducing the feature selection and adjustment that must be done manually in regression analysis.
4. Scalability: Machine learning algorithms are designed to work with large data sets and can be trained on such data. This offers an advantage over regression methods in analyzing and predicting large-scale data sets.
5. Customizable and extensible: Machine learning models can be adjusted to specific problems because they are customizable and extensible. This refers to adaptability to different types of data sets and various prediction problems.
6. Modelling complex relationships: Machine learning can model complex relationships and interactions between multiple inputs, resulting in more sophisticated and accurate predictions.

While regression analysis can be powerful and useful in certain situations, these advantages offered by machine learning often provide more effective solutions for a wider range of applications and challenging data analysis problems. In this study, models through R program were used to predict microbiota awareness.

## 3. Results

The study included participants who predominantly lived in nuclear families (86.6%) and in provinces (66.4%). A significant portion of the participants had mothers who were not employed (66.2%) and had a father who were employed (84.6%). Almost half of the participants reported having an income equal to their expenses (47.9%). The majority of participants reported not smoking (88.6%) and not consuming alcohol (77.1%). About one-third of the participants reported receiving information about nutrition (30.3%), with 30.3% reporting teachers as their source of that information. The consumption of fast food two to three times a week was reported by 34.4% of the participants, and 80.0% reported skipping meals, with breakfast being the most frequently skipped meal. The mean scores for the Microbiota Awareness Scale, Nutrition Literacy Scale, and Health Literacy Scale were 63.20 ± 11.46, 71.82 ± 14.67, and 91.38 ± 18.90, respectively. The mean age of the participants was 15.42 ± 1.32 years (Table 1).

**Figure 1:**
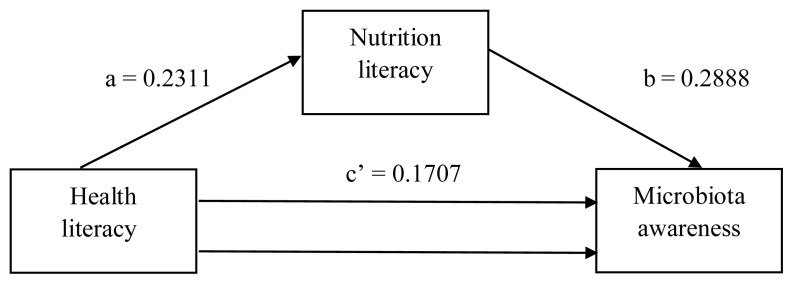
The mediating effect of the nutrition literacy variable on the effect of health literacy variable on the microbiota awareness variable. Statistical model of simple mediation analysis.

The total effect of the health literacy variable on nutritional literacy was 0.2311, with statistical significance at the 95% CI (p < 0.05). Furthermore, the lower limit CI (LLCI) and upper limit CI (ULCI) values (0.1775 and 0.2847, respectively) did not contain zero values between them, demonstrating the significance of this model. As seen in Table 2 and Figure 1, a one-unit increase in health literacy resulted in a 0.2311 unit increase in nutritional literacy.

In terms of the health literacy variable, the direct effect of the nutrition literacy variable on microbiota awareness was 0.2888, with statistical significance at the 95% CI (p < 0.05). LLCI and ULCI values of 0.2385 and 0.3392, respectively, also demonstrated the significance of this model (Table 2 and Figure 1).

The total effect of the health literacy variable on the microbiota awareness variable was 0.2374, which was statistically significant at the 95% CI (p < 0.05). The LLCI (0.1971) and ULCI (0.2778) values also demonstrated the significance of this model, as they did not include zero values between them (Table 2 and Figure 1).

Furthermore, in terms of the nutritional literacy variable, the direct effect of the health literacy variable on the microbiota awareness variable was 0.1707, which was also statistically significant at the 95% CI (p < 0.05). Nutrition literacy had a partial mediating role in the effect of health literacy on microbiota awareness (LLCI: 0.045 and LLCI: 0.0894). This statistical significance indicated partial mediation. In this case, the health literacy level was partially affected by the nutrition literacy level and had a partial effect on the microbiota variable. Finally, the indirect effect of nutrition literacy was statistically significant (LLCI: 0.0458 and ULCI: 0.0894) and played a partial mediating role in the interaction between these two variables. Health literacy influenced microbiota awareness directly and indirectly through nutrition literacy. The total effect of health literacy on microbiota awareness was larger than its direct effect, suggesting that nutrition literacy plays an important mediating role. Since the indirect effect was significant, some of the effect of health literacy on microbiota awareness was realized through nutritional literacy. These results suggest that increasing health literacy can increase microbiota awareness, both directly and indirectly through nutrition literacy (Table 2, Figure 1).

**Figure 2:**
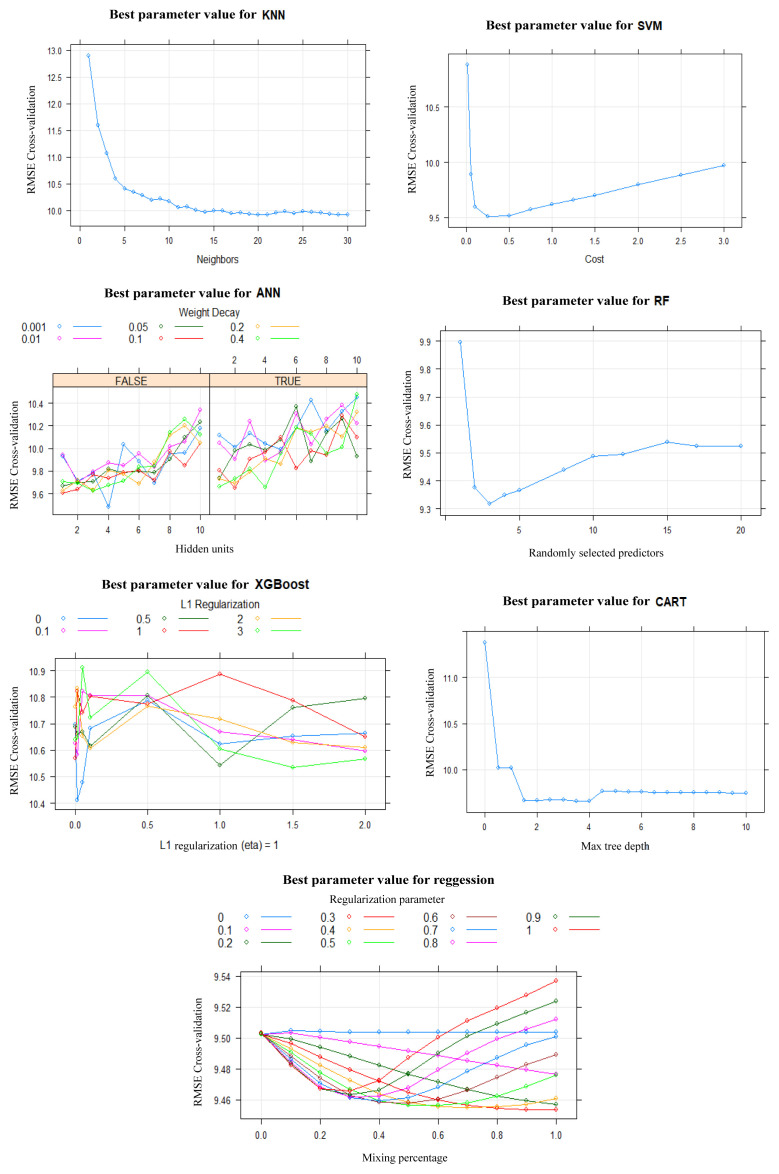
KNN, SVM, ANN, RF, XGBoost, CART, and regression algorithm models used for the estimation of the microbiota awareness variable and determining the best parameter value according to the train data.

Nutrition literacy, health literacy, family type, place of residence, mother’s occupation, father’s occupation, monthly income, smoking status, receiving information about nutrition, frequency of fast-food consumption, and meal skipping variables were used for the microbiota awareness estimation. In the construction of the estimation model, the most accurate parameter values for seven different algorithms were determined using 10-fold cross validation. This method divided the dataset into 70% for training (519 observations) and 30% for testing (220 observations), ensuring a balanced approach for both model training and evaluation. The training phase focused on fine-tuning the algorithms’ parameters to achieve the highest possible accuracy, and the performance outcomes are illustrated in Figure 2. This structured approach not only facilitated the optimal adjustment of parameters but also allowed for a comprehensive comparison across algorithms, emphasizing the importance of precision in parameter selection to enhance the model’s predictive capability. By employing 10-fold cross validation and strategically splitting the data, the algorithms’ settings were efficiently optimized, aiming to improve the reliability and validity of the model’s predictions. The visualization of the results in Figure 2 provides a clear comparison of the algorithms’ performance, highlighting the effectiveness of the chosen methodology in identifying the most suitable parameter values for each algorithm, thereby ensuring the model’s accuracy and generalizability (Figure 2).

**Figure 3:**
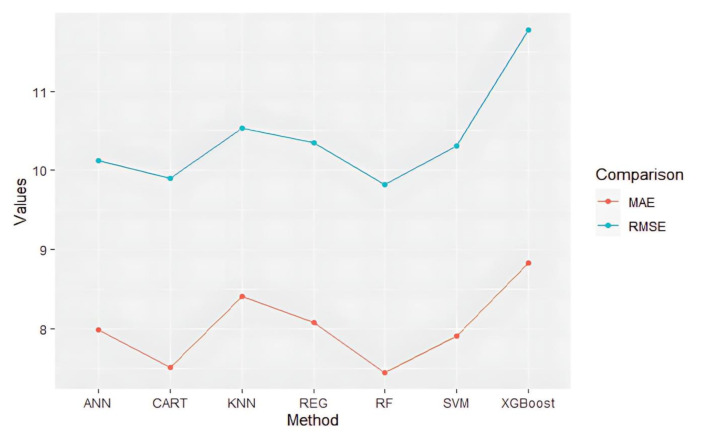
Metric values of the methods based on the estimation of the test data.

When analyzed with comparison metrics (root-mean-squared error and mean absolute error) for these best parameter values, Figure 3 was obtained.

**Figure 4:**
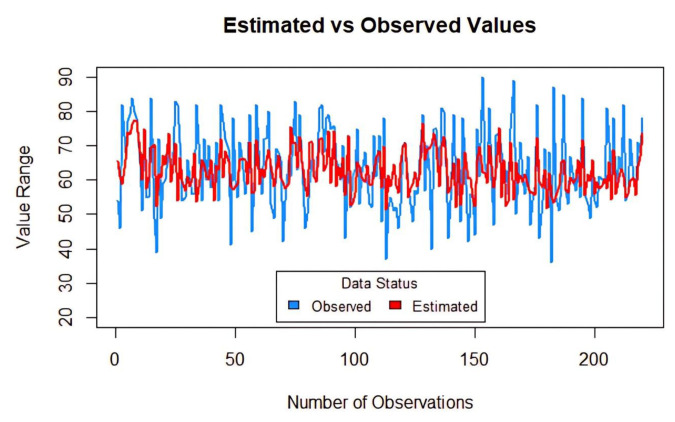
Microbiota awareness prediction with the RF method.

A prediction table was created for the random forest (RF) method (Figure 4).

**Figure 5:**
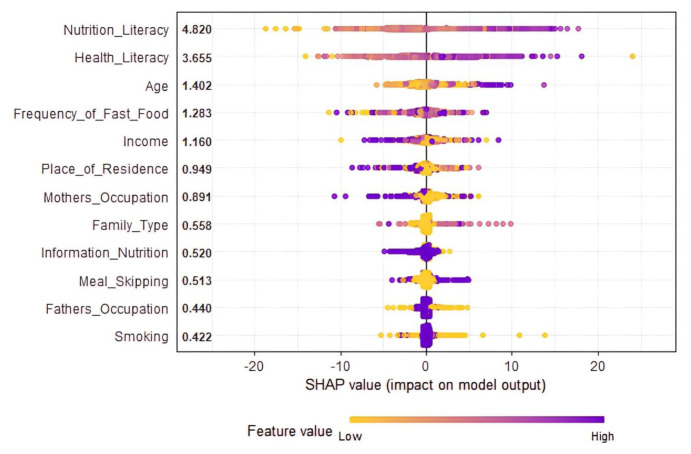
Determining the variable contributions to the microbiota awareness estimation model with Shapley values.

In the prediction model, the efficacy of all the variables was assessed across various machine learning algorithms to determine their impact on the model’s performance. The significance of each variable was quantified using Shapley values, derived from Shapley additive explanations (SHAP), ensuring a standardized measure of contribution towards the predictive accuracy of the model. SHAP values offer a detailed view into the role each variable plays in the model’s predictions, highlighting the importance of these variables in an unbiased manner against the performance criterion. When analyzing the SHAP values for the best-performing model, it was crucial to examine these values to maintain an objective comparison of variable significance. This approach helps in identifying the contribution or importance of each variable within the model’s prediction process. The analysis revealed that among all the variables considered, nutrition literacy emerged as the most critical predictor for the microbiota awareness variable, indicating its pivotal role in the model. This insight underscores the value of incorporating nutrition literacy into the model to enhance the predictability and understanding of microbiota awareness, highlighting its significant influence on the overall model performance (Figure 5).

## 4. Discussion

This study revealed that the level of health literacy was positively associated with the level of nutritional literacy (p < 0.05). Limited studies in the literature have compared the health and nutritional literacy levels of adolescents. Kırşan and Özcan [31] found a weakly positive and significant (p < 0.001) relationship between health literacy and nutritional literacy scores. Similarly, SaeidiFard et al. [32] examined the relationship between health literacy, nutritional literacy, and sun exposure, and found that the majority of the participants had high health literacy levels, while their nutritional literacy levels were low.

Although the current study differs from the existing literature, it has been reported that dietary habits play a crucial role in explaining the differences in individuals’ health literacy levels. While no studies have demonstrated individuals’ correct understanding of messages related to protecting, improving, and treating health, such as nutrition education and medical nutrition therapy, it is known that individuals with healthy diets have higher health literacy levels [33–35].

In the current study, the level of nutritional literacy increased the level of microbiota awareness in the presence of the health literacy variable (p < 0.05). In other words, nutritional literacy had a positive impact on microbiota awareness. While there are no studies comparing nutritional literacy and microbiota awareness specifically, previous research has shown that education and knowledge level are positively associated with microbiota awareness [36,37]. Similarly, individuals with a medical degree [38] and students who have taken a microbiology course at university [39] have been found to have greater knowledge of prebiotics and microbiota. This may be because consumers are expected to have a higher general knowledge level as a result of researching or reading the prospectus before starting to use a nutritional supplement. However, the high level of knowledge does not always lead to the behavior of using that product.

Furthermore, the current study found that the level of health literacy also increased the level of microbiota awareness (p < 0.05). This confirmed the expected indirect impact of health literacy on microbiota awareness. While there are no studies comparing these two parameters directly, a study conducted in Iran found that although the majority of participants were aware of the presence of beneficial microorganisms in probiotic dairy products, they did not consume enough milk-based probiotics [37]. It is important to note that while knowledge and education level are positively associated with awareness, this does not necessarily translate into a change in behavior [31]. Therefore, more longitudinal and experimental approaches are needed to explore the complex relationship between health literacy, nutritional literacy, and microbiota awareness.

In the present study, health literacy influenced microbiota awareness directly and indirectly through nutrition literacy. The total effect of health literacy on microbiota awareness was larger than its direct effect, suggesting that nutrition literacy plays an important mediating role. Since the indirect effect was significant, some of the effect of health literacy on microbiota awareness was realized through nutritional literacy. These results suggest that increasing health literacy can increase microbiota awareness both directly and indirectly through nutrition literacy. Moreover, microbiota awareness increased as health literacy and nutrition literacy increased. In the machine learning approach prediction, the most important variables affecting microbiota awareness were health literacy and nutritional literacy. Developing appropriate intervention methods to improve individuals’ health and nutritional literacy could also improve their awareness of microbiota. To design effective interventions, it is crucial to consider the relationship between the media, food, health, and education system. It is recommended to conduct stakeholder and needs analysis, determine objectives, ground on scientific theories, approaches, and models, use appropriate training methods, and evaluate and monitor the interventions’ effectiveness. Longitudinal studies on microbiota awareness are recommended.

## Figures and Tables

**Table 1:** Descriptive characteristics of the participants (n = 739).

Demographic features		n	%
Family type	Nuclear	640	86.6
	Extended	90	12.2
	Broken	9	1.2
Place of residence	Province	491	66.4
	District	126	17.1
	Village	122	16.5
Mother’s employment status	Not working	489	66.2
	Working	250	33.8
Father’s employment status	Not working	114	15.4
	Working	625	84.6
Monthly income	Less than my expenses	199	26.9
	Equal to my expenses	354	47.9
	More than my expenses	186	25.2
Smoking status	Yes	84	11.4
	No	655	88.6
Nutritional information status	Yes	570	77.1
	No	169	22.9
Source of nutritional information	Radio-TV	99	13.4
	Teacher	224	30.3
	Health personnel	119	16.1
	Conference, seminar	83	11.2
	Book, magazine	45	6.1
Frequency of fast food consumption	Never	50	6.8
	Every day	225	30.4
	2–3 times a week	254	34.4
	Once a week	127	17.2
	Once a month	83	11.2
Meal skipping status	Yes	591	80.0
	No	148	20.0
Most skipped meal	Morning	376	50.9
	Noon	176	23.8
	Evening	39	5.3
Scales	*X̄* **±** SD	Min	Max
Microbiota Awareness Scale Total Score average	63.20 **±** 11.46	35	93
Nutrition Literacy Scale Total Score average	71.82 **±** 14.67	34	108
Health Literacy Scale Total Score average	91.38 **±** 18.90	45	125
Age (years)	15.42 **±** 1.32	14	19

**Table 2:** Mediation analysis results: the role of nutrition literacy in the relationship between health literacy and microbiota awareness.

Model	Coefficient	Standard error	t	p-value	LLCI	ULCI
Health literacy nutrition literacy (total effect)	0.2311	0.0273	8.4627	0.0001	0.1775	0.2847
Nutrition literacy microbiota awareness (direct effect)	0.2888	0.0256	11.2704	0.0001	0.2385	0.3392
Health literacy microbiota awareness (total effect)	0.2374	0.0206	11.5483	0.0001	0.1971 (bootstrap)	0.2778 (bootstrap)
Health literacy microbiota awareness (direct effect)	0.1707	0.0199	8.5763	0.0001	0.1316 (bootstrap)	0.2097 (bootstrap)
Nutrition literacy microbiota awareness (indirect effect)	0.0667	0.0112	-	-	0.0458 (bootstrap)	0.0894 (bootstrap)

## Footnotes

1 Turkish Statistical Institute (2023). Address-Based Population Registration System Results [online]. Website https://data.tuik.gov.tr/Bulten/Index?p=Adrese-Dayal%C4%B1-N%C3%BCfus-Kay%C4%B1t-Sistemi-Sonu%C3%A7lar%C4%B1-2023-49684&dil=1 [accessed 02 February 2024].
